# Patrolling the nucleus: inner nuclear membrane-associated degradation

**DOI:** 10.1007/s00294-019-00971-1

**Published:** 2019-04-24

**Authors:** Christine J. Smoyer, Sue L. Jaspersen

**Affiliations:** 1 Stowers Institute for Medical Research, 1000 E. 50th Street, Kansas City, MO 64110 USA; 2 Department of Molecular and Integrative Physiology, University of Kansas, Medical Center, Kansas City, KS 66160 USA; 3 Present Address: Department of Molecular and Cellular Biology, University of California, Davis, CA 95616 USA

**Keywords:** Inner nuclear membrane, ERAD, INMAD, Asi complex, Protein quality control

## Abstract

Protein quality control and transport are important for the integrity of organelles such as the endoplasmic reticulum, but it is largely unknown how protein homeostasis is regulated at the nuclear envelope (NE) despite the connection between NE protein function and human disease. Elucidating mechanisms that regulate the NE proteome is key to understanding nuclear processes such as gene expression, DNA replication and repair as NE components, particularly proteins at the inner nuclear membrane (INM), are involved in the maintenance of nuclear structure, nuclear positioning and chromosome organization. Nuclear pore complexes control the entry and exit of proteins in and out of the nucleus, restricting movement across the nuclear membrane based on protein size, or the size of the extraluminal-facing domain of a transmembrane protein, providing one level of INM proteome regulation. Research in budding yeast has identified a protein quality control system that targets mislocalized and misfolded proteins at the INM. Here, we review what is known about INM-associated degradation, including recent evidence suggesting that it not only targets mislocalized or misfolded proteins, but also contributes to homeostasis of resident INM proteins.

## Introduction

Long before the discovery of DNA, the cell nucleus was depicted in the drawings of botanists and zoologists as the first organelle, and it was postulated to be a ubiquitous feature of cells (Rosner et al. 2005). Today, we understand that nuclei are the defining feature of eukaryotic cells, containing the majority of genetic material, which is needed for cell growth, division and function. The NE is composed of two lipid bilayers: an outer nuclear membrane (ONM), which is contiguous with the endoplasmic reticulum (ER), and an inner nuclear membrane (INM), which is separated from the ONM/ER by nuclear pore complexes (NPCs). Early work in animal cells showed that proteins localized to the INM such as lamins give the nucleus its unique round shape and rigid structure, while proteins such as lamin B receptor, barrier to autointegration factor (BAF), and the lamin–emerin–man1 (LEM) domain proteins are involved in the organization of chromosomes, influencing gene expression, DNA replication and repair (reviewed in Barton et al. 2015; Dittmer and Misteli 2011; Gerace and Tapia 2018; Gruenbaum and Foisner 2015).

Changes in nuclear morphology, as found in cells with defects in levels or distribution of INM components, are linked to aging, age-related diseases, and cancer. Loss or mutation of both INM and ONM proteins are related to a wide spectrum of diseases, while misfolding and aggregation of nuclear proteins are a feature of neurodegenerative diseases (reviewed in Burke and Stewart 2014; Dahl et al. 2006; Janin et al. 2017; Woulfe 2008). Despite these correlations and connections with human pathology, we are just beginning to understand mechanisms that contribute to INM protein homeostasis. Here, we review what is known about NE composition and its regulation, with an emphasis on roles for inner nuclear membrane-associated degradation (INMAD) in NE homeostasis. Understanding mechanisms such as INMAD that control the distribution and concentration of NE components will shed light on NE functions such as chromosome organization, nuclear positioning and DNA repair.

## Towards a nuclear membrane proteome

In 2003, the first set of nuclear envelope transmembrane (NET) proteins from rat liver nuclei was published, expanding on the list of thirteen known integral membrane proteins in animals (Schirmer et al. 2003). Interestingly, many of the NETs were encoded by genes linked to various dystrophies, highlighting the importance of the NE in human disease. In other tissues, the NET proteome showed a mere 16% overlap with that of liver, with many of the shared proteins also showing differences in abundance between tissues (Korfali et al. 2012; Wilkie et al. 2011). Adding to the complexity of NETs, a report from mammalian cells estimated that 40% of cellular proteins are shared between organelles (Foster et al. 2006). While this is perhaps not surprising given the continuity between the ER and ONM, these studies illustrate the complexity of determining the composition of the INM using biochemical approaches. Electron microscopy (EM) has been the gold standard for determining if proteins reside at the INM. However, EM is suited to characterization of individual proteins, and it provides a snapshot of protein distribution because only a handful of cells are typically examined. Recent work in yeast illustrated the promise of split GFP to identify and study proteins at the INM (Smoyer et al. 2016)—a strategy that has now been adapted to mammalian systems as well (Tsai et al. 2019). In a genome-wide screen of all known or predicted membrane proteins in *Saccharomyces cerevisiae*, 411 (roughly 6% of the yeast genome) showed evidence of INM access. Of these, approximately 35% of proteins detected at the INM were shared with the ER (Smoyer et al. 2016), consistent with observations in higher eukaryotes that the INM proteome overlaps with other organelles.

How specific membrane proteins are targeted and retained at the INM of certain cells is an important problem that the field is just beginning to unravel. In theory, one can envision at least two major mechanisms that contribute to INM composition: access and surveillance. We will briefly review what is known about INM transport, as this is the subject of a number of reviews (Katta et al. 2014; Lusk et al. 2007; Satoh et al. 2018; Ungricht and Kutay 2015; Zuleger et al. 2012). Then, we will focus this review on the emerging role of protein quality control in shaping the INM landscape.

## Getting to the INM

**Fig. 1:**
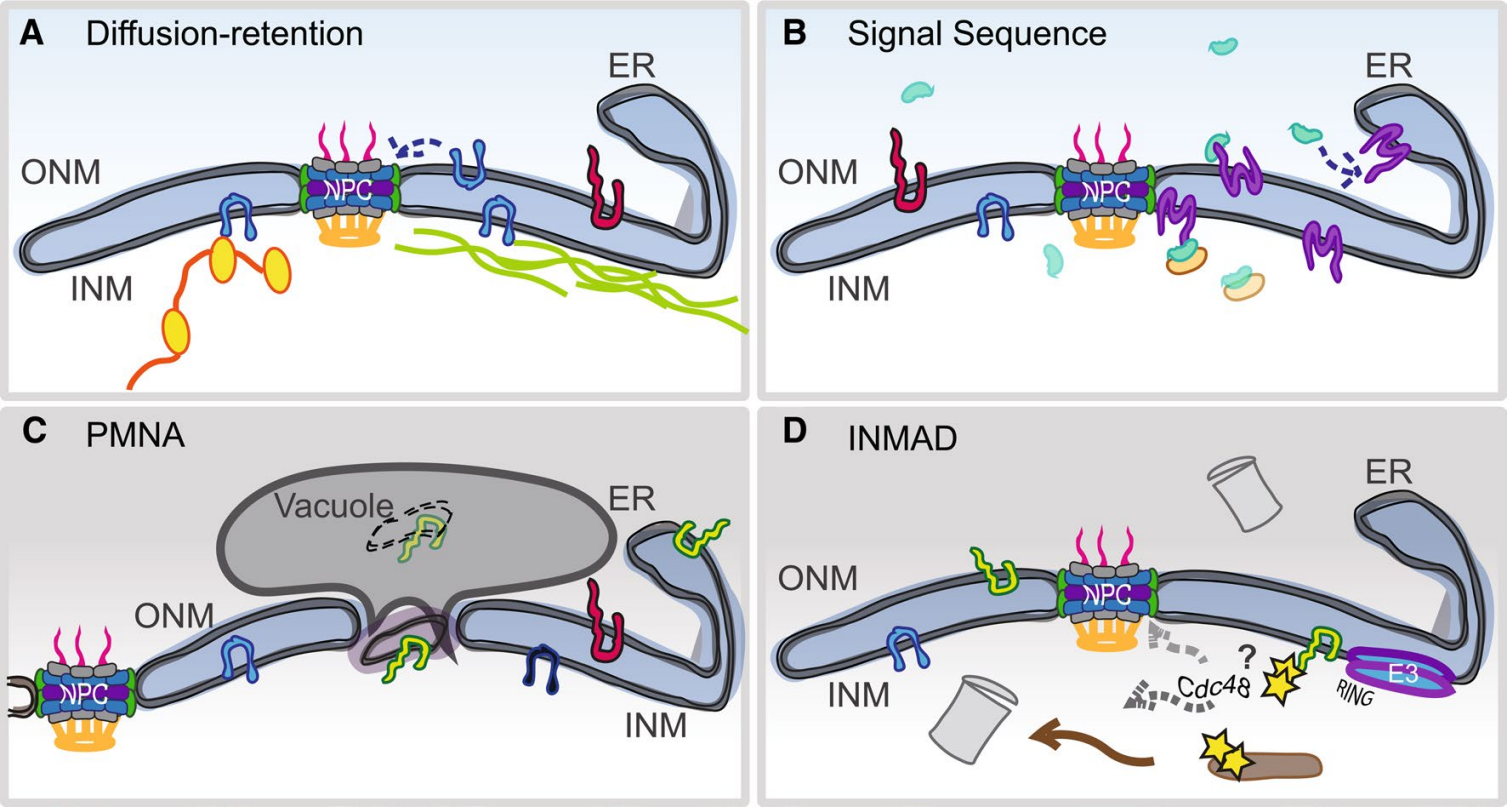
Post-translational mechanisms of INM homeostasis. **a** The diffusion retention model supports the idea that a NET (blue) is able to freely diffuse in and out of the nucleus as long as its cytoplasmic/nucleoplasmic domain is small enough to travel through the peripheral channel of NPCs (~ 60 kDa). NETs accumulate inside the nucleus by tethering to other nuclear factors such as chromatin (yellow) and/or lamins (green). **b** Using a sequence to signal a truncated version of karyopherin-α (green), an INM-bound NET (purple) is translocated through an NPC, followed by release of the karyopherin by association with Nup50/Nup2 (orange) or by other mechanisms. **c** Piecemeal nuclear autophagy (PMNA) occurs in the nuclei of yeast under nutrient deprivation, when sections of the INM are pinched off into the vacuole (yeast equivalent of the lysosome). PMNA is not known to occur in higher eukaryotes although there is evidence of autophagy of NE proteins (Dou et al. 2015). **d** Similar to ERAD, the INMAD pathway involves the ubiquitination of targets (yellow) by an E3 ligase containing a RING domain (blue–purple). Degradation of soluble targets may occur inside the nucleus by nuclear-localized proteasomes (gray). For membrane-bound targets, the Cdc48 AAA-ATPase may be involved in removal of the protein from the INM for degradation. Alternatively, retrotranslocation of the targets back to the ER for removal and processing by cytoplasmic ERAD machinery may occur

Two major pathways for INM transport have been described (Fig. 1a, b). Similar to transport of soluble cargos greater than ~ 40–60 kDa, sequence-based targeting involves a motif on an extraluminal region of the INM cargo that is recognized by a karyopherin, which mediates movement of the protein through the NPC central channel to the INM. The diffusion–retention pathway is thought to be used by proteins that contain cytoplasmic/nucleoplasmic domains below a certain size threshold (typically ~ 60 kDa). These membrane proteins are free to diffuse in and out through lateral NPC channels. Binding to lamins, chromatin or other nuclear proteins retain some proteins at the INM, leaving non-INM proteins free to diffuse back out. The majority of support for the sequenced-based pathway has come from yeast and other model organisms investigating transport of a single INM cargo, whereas diffusion–retention more readily explains the transport requirements of multiple NETs and other INM components in mammalian cells (Boni et al. 2015; Burns and Wente 2012; Ellenberg et al. 1997; Hasan et al. 2006; Katta et al. 2014; King et al. 2006; Liu et al. 2010; Meinema et al. 2013; Smoyer et al. 2016; Soullam and Worman 1995; Tapley et al. 2011; Wente and Rout 2010). Our recent genome-wide analysis of INM localization in yeast largely supports the diffusion–retention model, as we found no enrichment for sequences but rather a strong enrichment for domain size in proteins able to access the INM (Smoyer et al. 2016).

The idea that proteins below a size threshold are able to diffuse through NPCs suggests that many small membrane proteins should be found at least transiently or at low levels at the INM. However, this model whereby the nuclear interior is freely accessible to small proteins via diffusion is a Pandora’s box, opening the gates to the genome to any protein from any host that is small enough to sneak past. One possible solution to the free diffusion enigma is to evolve rigorous quality control mechanisms that regulate and patrol the INM. Mechanisms to turn over proteins at the INM could remove harmful or toxic factors. In addition, these same pathways could also contribute to changes in INM composition that occur during the cell cycle, in development and differentiation and during disease.

## Mechanisms of protein quality control

Protein quality control systems provide a safeguard for many cellular processes and play an important role in organelle homeostasis by removing misfolded or damaged proteins. Eukaryotic cells have evolved multiple interconnected pathways to cope with the burden of misfolded or damaged proteins, including chaperone-dependent refolding or sequestration, association with heat shock proteins, degradation by the ubiquitin–proteasome system and autophagic destruction in the lysosome or vacuole, the yeast equivalent organelle (Brodsky 2012; Enenkel 2018; Park et al. 2009; Roberts et al. 2003; Webster and Lusk 2016; Zattas and Hochstrasser 2015).

One of the best studied examples of membrane–protein quality control pathways is that of the ER-associated degradation (ERAD) system. Defects in insertion, folding, assembly or post-translational modification of membrane proteins lead to poly-ubiquitination of the damaged protein. Two conserved E3 ligases, Doa10/MARCH6/TEB4 and Hrd1/SYVN1, respond to lesions in the cytosolic or luminal/membrane regions of ER proteins, respectively. ERAD targets are likely degraded in the cytoplasm by the 26S proteasome (reviewed in Brodsky 2012; Enenkel 2018; Romisch 2005; Zattas and Hochstrasser 2015). How membrane proteins are removed or retrotranslocated across the ER membrane is an area of active research. Current models include the Sec61 translocon and/or the action of the AAA-ATPase Cdc48 (Romisch 2005; Stolz et al. 2011; Ye et al. 2017).

Given that proteins diffuse to the NE from the ER, it seems like a similar robust quality control system would exist to ensure the integrity of the nucleus. However, examination of protein stability in rats using isotope labeling suggested that NPCs and INM proteins such as lamins are extremely long-lived, leading to the general idea that the NE proteome is stable, with little protein turnover (Savas et al. 2012). This view of a stable NE proteome is beginning to evolve. Recently, a NE surveillance pathway was described that utilizes the LEM domain protein Heh2 along with Vps4 and the ESCRT-III machinery to recognize aberrant NPC assembly intermediates and to confine these aggregates to a Storage for Improperly assembled Nuclear pore Complexes (SINC) compartment (Webster et al. 2014, 2016). How the SINC is turned over is not well understood; one possibility is that a region of the NE is pinched off, similar to piecemeal nuclear autophagy (PMNA) that occurs under nutrient deprivation, removing entire sections of the nucleus and NE (Fig. 1c). While autophagy at the nucleus was first observed in yeast (Roberts et al. 2003), recent work revealed that nuclear lamins in mammals are regulated by autophagy as well (Borroni et al. 2018; Dou et al. 2015; Lu and Djabali 2018; Luo et al. 2016).

**Fig. 2:**
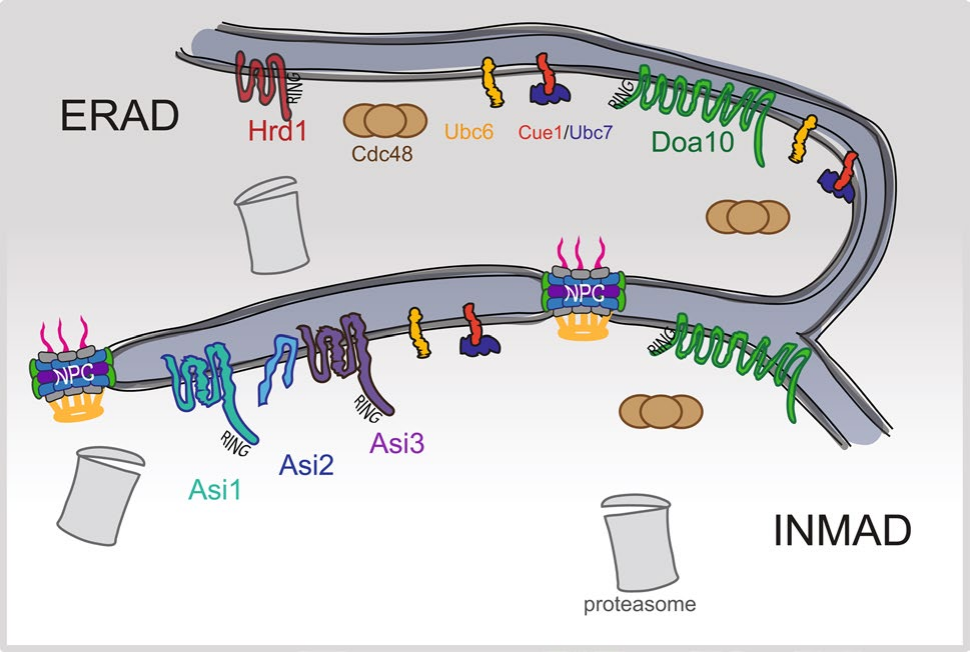
INMAD is an extension of ERAD. Membrane-bound E3 ligases of budding yeast are depicted at the ER only (Hrd1, dark red), at the ER and INM (Doa10, green) and at the INM only (Asi complex, green–blue–purple). The Asi complex is composed of the RING domain-containing proteins Asi1 and Asi3, as well as an accessory protein, Asi2. ERAD components, such as the E2 conjugating enzymes Ubc6 (yellow) and Ubc7 (blue) as well as the AAA-ATPase Cdc48 (brown) participate in the targeting of substrates to proteasomes (gray) for degradation. These components are also found inside the nucleus and likely contribute to INMAD. Ubc6 and Ubc7 have been shown to be associated with Asi1 and Asi3 through bimolecular fluorescence complementation (Khmelinskii et al. 2014). Proteasomes and Cdc48 have also been localized to the nucleus (Chen et al. 2011; Gallagher et al. 2014)

Given the continuity between the ONM and ER, ERAD likely surveys ONM components. It is possible that additional mechanisms may exist since certain membrane proteins are tethered specifically in the ONM through luminal interactions with INM proteins, such as the Klarsict-ANC-1-Syne-1 homology (KASH) domain proteins that form a luminal linker of nucleoskeleton and cytoplasm complex (LINC) with Sad1-UNC-84 (SUN) domain-containing proteins. It is unclear if ERAD can clear proteins from the INM due to the separation of ONM/ER by NPCs. In yeast, INMAD is thought to ensure protein quality control within the nucleus (Fig. 2). Three E3 ligases are involved in the INMAD pathway: Doa10, Asi1 and Asi3. Given the role of Doa10 in the ERAD pathway, and the evidence of Doa10 in turnover of the MATα2 transcription factor, INMAD has been proposed to be an extension of ERAD at the INM (Swanson et al. 2001). However, as we discuss below, INMAD has additional roles beyond protein quality control in shaping the INM landscape.

## The Asi complex and INMAD

Yeast cells utilize a signal transduction pathway to control the activity of two soluble latent transcription factors, Stp1 and Stp2. In response to amino acid availability, these factors are cleaved, allowing them to enter the nucleus to regulate transcription of multiple amino acid permeases (Andreasson et al. 2006; Andreasson and Ljungdahl 2002; Wang et al. 1992). *ASI1*, *ASI2* and *ASI3* were isolated in a screen for mutants (amino acid sensitive independent) that short circuit this pathway, allowing uncleaved Stp1 and Stp2 to activate transcription in the absence of amino acid induction (Boban et al. 2006; Forsberg et al. 2001; Zargari et al. 2007). *ASI1* and *ASI3* encode two related RING finger E3 ligases that localize to the INM of budding yeast (Zargari et al. 2007). Asi2 does not contain a domain conferring a ligase function but is also present at the INM. Asi1, Asi2 and Asi3 associated in a large Asi complex, although it is important to note that Asi2 is not essential for formation of the Asi1–Asi3 heterodimer or its activity (Foresti et al. 2014). Instead, Asi2 is thought to be an adaptor or recognition factor needed for ubiquitination of certain substrates (discussed below). Deletion of each gene renders cells insensitive to amino acid sensor signaling (Zargari et al. 2007). The fact that double and triple mutants are not additive supports the idea that the three proteins form a single large Asi complex.

As part of amino acid sensing, the Asi complex removes inappropriately targeted soluble transcription factors from the nucleus through ubiquitination; the targets are destroyed within the nucleus by nuclear-localized 26S proteasomes (Boban et al. 2006; Zargari et al. 2007). Thus, as an INM E3-ligase, the Asi complex is perhaps ideally suited to INM surveillance, possibly targeting mislocalized or damaged membrane proteins for destruction as part of INMAD. The Asi complex along with Doa10 also could target proteins that diffuse into the INM, helping to shape the INM proteome.

To identify substrates of the Asi complex, the Knop lab employed a tandem fluorescent timer, which exploits the fact that GFP and mCherry have different maturation times in yeast (Khmelinskii et al. 2014). This tool can be used as a proxy for protein half-life, comparing stability in wild-type cells or in mutants. A proteomics approach was also used to identify putative targets (Foresti et al. 2014). The sterol biosynthesis proteins Erg11 and Nsg1 and the vacuolar proteins Vcx1, Vtc1, and Vtc4 were identified in these genome-wide screens, and all were shown to increase in protein level as well as accumulate at the nuclear periphery upon mutation of *ASI1* and to a lesser extent with that of *ASI3* (Foresti et al. 2014; Khmelinskii et al. 2014). At the time, this result was interpreted as a sign of protein mistargeting since lipid biosynthesis, for example, was thought to occur in the ER. Recently, metabolic activity has been observed at the yeast INM suggesting that at least some lipid enzymes could be resident INM proteins (Romanauska and Kohler 2018). Thus, some of these putative targets may not be mistargeted proteins but instead are INM components and bonafide Asi complex substrates regulated in much the same way as other native INM proteins (discussed below).

Other players in INMAD were identified by genetic or protein interaction studies with Asi1, Asi2 or Asi3. Attachment of ubiquitin is catalyzed by one of two E2 ubiquitin-conjugating enzymes, Ubc6 or Ubc7, in a reaction involving the Asi complex (Foresti et al. 2014; Khmelinskii et al. 2014). The ERAD E3 ligase Doa10 also localizes to the INM, where it mediates the turnover of a soluble transcription factor as well as Asi2 (Boban et al. 2014; Swanson et al. 2001). A major outstanding question is how the INMAD recognizes its substrates—is there an INM degron or does the INMAD simply detect protein misfolding? The observation that a mutant form of the ER translocon Sec61 deliberately mistargeted to the INM using a nuclear localization sequence was also partially stabilized by loss of *ASI1* suggests that the Asi complex, and by extension INMAD, may recognize misfolding (Foresti et al. 2014).

## INMAD and INM homeostasis

To better understand the impact that protein quality control pathways play in INM composition, we recently examined the distribution of all C-terminally tagged *Saccharomyces cerevisiae* membrane proteins in wild-type cells and INMAD, ERAD and vacuolar proteolysis mutants (Smoyer et al. 2019). As anticipated, deletion of the Asi complex had a more pronounced effect on the INM compared to mutants in vacuolar or ERAD pathways. This approach confirmed eight of twelve previously identified INMAD substrates, including the vacuolar transferase complex subunit Vtc1 that is thought to be mistargeted to the INM due to protein tagging, the Rab GTPase interacting protein Yip4, the plasma membrane transporter Zrt2, inositolphosphotransferase Ipt1, and Irc23, a protein of unknown function that is linked to DNA damage (Khmelinskii et al. 2014). One of the most surprising results was that the levels and/or distribution of native INM components was also affected by loss of INMAD, including Asi2, the LEM domain protein Heh2 and the nucleoporins Pom33 and Pom34 (Smoyer et al. 2019). Analysis of these native INM substrates has expanded our understanding of the role INMAD plays in INM homeostasis.

It seems clear that INMAD components themselves are targets of protein quality control pathways, although interestingly, each subunit of the Asi complex is regulated in a specific manner. A comparison of protein half-lives shows that in wild-type yeast grown in rich media, the stability of Asi1, Asi2 and Asi3 is approximately 30, 45 and 90 min, respectively (Boban et al. 2014; Pantazopoulou et al. 2016). One trivial explanation for the different half-lives is that cells simply turn over monomeric forms of each protein as a quality measure to ensure the INM contains functional, fully assembled Asi complex. However, the half-lives of Asi1 and Asi2 are not affected by deletion of other complex members, making this less likely a possibility. In addition, Asi3 is considerably more stable than Asi2 or Asi1. As a paralog of Asi1 that arose during the yeast whole genome duplication, it is possible that it lost sequences important for turnover or acquired residues that protect it from ubiquitination and/or proteolysis. Analysis of the trans-acting factors needed for Asi1 and Asi2 turnover showed that both require Ubc7 and the nuclear 26S proteasome, but only Asi2 poly-ubiquitination was Doa10 dependent (Boban et al. 2014). A panel of E3 ligases was screened for roles in Asi1 stability and none was found to regulate Asi1 (Pantazopoulou et al. 2016). One intriguing candidate that was absent from this list was the anaphase promoting complex (APC), a multi-subunit E3 ligase known from its role in cell cycle control. The APC was recently reported to control the ubiquitination and turnover of the yeast SUN protein Mps3 at the INM (Koch et al. 2019), so it is possible that it could also control Asi1 poly-ubiquitination and degradation. The fact that Asi1 turnover is affected by mutations in the AAA-ATPase Cdc48 suggests that membrane proteins are extracted from the INM in much the same way they are from the ER membrane during ERAD (Pantazopoulou et al. 2016), however, follow-up on this result is needed to understand globally how proteins are removed from the INM.

**Fig. 3:**
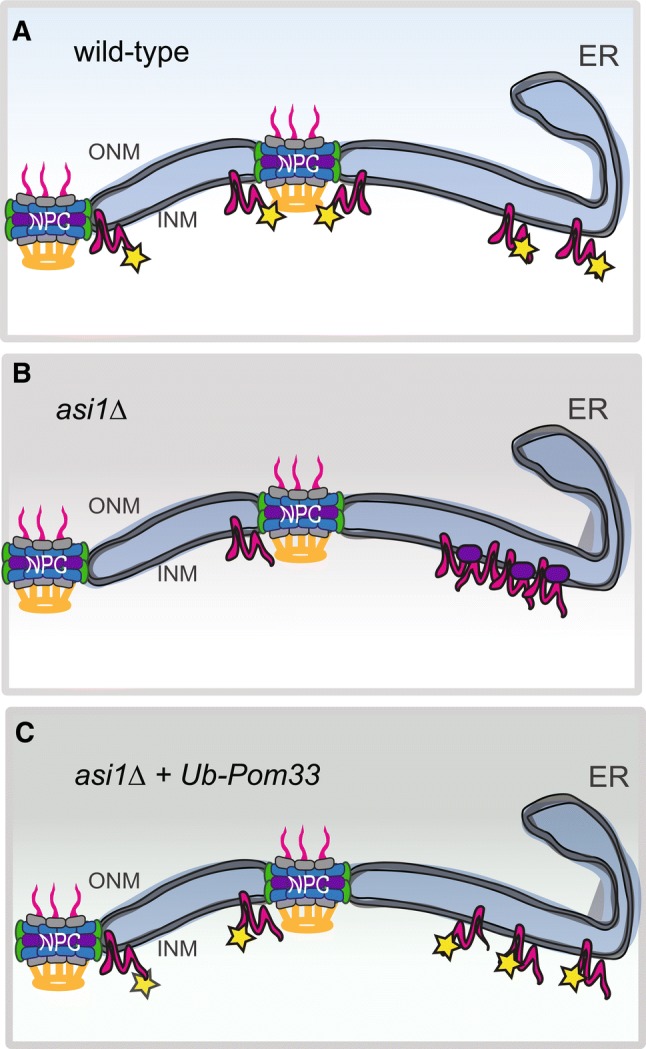
INMAD ubiquitination of Pom33 drives its INM distribution. **a** A pool of Pom33 (magenta) is ubiquitinated (yellow), and contributes to its INM distribution. **b** When *ASI1* is deleted, Pom33 aggregates into one or more foci at the INM. These foci colocalize with a subset of Nups, such as Nup188 (purple). **c** When a single ubiquitin was added back to Pom33 at its N-terminus, Pom33 re-distributed in the INM, even in the absence of Asi1

Pom33 and its paralog Per33 are also targets of INMAD. Pom33 ubiquitination is Asi1, Asi2 and to a lesser degree Asi3 dependent, although a chain of poly-ubiquitinated protein was not observed (Smoyer et al. 2019). Importantly, Pom33 ubiquitination did not affect protein stability but rather altered the distribution of Pom33 at the NE, providing evidence that INMAD plays a role in proteostasis of wild-type INM proteins (Fig. 3) (Smoyer et al. 2019). Attachment of ubiquitin to Pom33 appears to play a role in protein targeting or distribution in much the same way that mono-ubiquitination of proteins is thought to expose cryptic localization or trafficking instructions at the NE or throughout the secretory pathway (Chen and Mallampalli 2009; Li et al. 2003; Lohrum et al. 2001; Nie et al. 2007). Unmodified Pom33 that is present in E2 or E3 ligase mutants of INMAD localizes to one or two large nuclear puncta in addition to the INM signal seen in wild-type cells. As Pom33 is a component of NPCs, we suspected the aggregates would contain other nucleoporins as well as components of the SINC. Colocalization analysis indicates that at least some additional NPC components are present in the puncta, although it does not seem that they are enriched for a particular NPC assembly intermediate nor do puncta contain SINC components. An important future question is to further characterize the molecular nature of this new NE compartment.

Could identification of Pom33 as an INMAD substrate, albeit a non-canonical target, be an inroad to understand INM homeostasis in other eukaryotes? Could it be a tool to elucidate the mechanism used to identify native versus foreign INM proteins? While the Asi complex is only present in budding yeast, Pom33/Tts1/TMEM33 is present throughout eukaryotes where it plays roles in NPC distribution, biogenesis and/or stability as well as nuclear envelope remodeling (Chadrin et al. 2010; Zhang and Oliferenko 2014). Analysis of Tts1 domains needed at the nuclear pore suggested a key role for its C-terminal amphipathic helix—a similar region also needed for both stability and NPC localization of Pom33 in budding yeast (Floch et al. 2015; Zhang and Oliferenko 2014). We suspect that multiple lysine residues within the C-terminal domain are ubiquitinated, suggesting it is an INMAD degron. An amphipathic helix is also thought to play a role in Asi2 degradation and Doa10 substrate recognition (Boban et al. 2014; Ravid et al. 2006), pointing to the possibility that these motifs are in some way part of INM surveillance to ensure NE homeostasis.

While one might argue that INMAD is only essential for fungi and plants that undergo a closed mitosis where the INM remains intact throughout cell division, it is important to note most cells in an adult animal are post-mitotic. Our work in yeast suggests that INM ubiquitination does more than regulate protein turnover, playing roles in the distribution of native INM components. The E3 ligases RNF123 and HECW2 have been identified in the degradation of several NE-associated proteins, including lamin B1, PCNA, LAP2 and emerin (Khanna et al. 2018; Krishnamoorthy et al. 2018). Interestingly, when nuclear export was blocked using leptomycin B, PCNA was still degraded by HECW2, indicating that degradation is occurring in the nucleus (Krishnamoorthy et al. 2018).

## Conclusion

Alterations in nuclear morphology have been correlated with both aging and cancer, and molecular and genetic studies have shown that mutations in lamins, nucleoporins, LEM and SUN domain-containing proteins and other NE components are related to a wide spectrum of diseases (reviewed in Burke and Stewart 2014; Dahl et al. 2006; Janin et al. 2017; Woulfe 2008). Understanding how the INM proteome is regulated by protein targeting and quality control pathways will provide insight into how the loss, mutation, or aggregation of NE proteins leads to nuclear dysfunction as well as elucidate new strategies for repairing or reversing damage. While analyzing protein regulation at the INM has been a challenge, split GFP has emerged as powerful tool for studying INM dynamics in yeast and more recently mammalian systems (Mashahreh et al. 2019; Smoyer et al. 2016, 2019; Tsai et al. 2019). Our work, along with that of others, has led to the following model. Most membrane proteins reach the INM by diffusion, while a few, such as SUN and LEM domain-containing proteins, are actively transported. Mistargeted and misfolded proteins are turned over at the INM by INMAD in yeast and likely by other unidentified E3s in metazoans. The INMAD system also plays a role in homeostasis of native INM components, controlling NE distribution through ubiquitination. Future work aimed at dissecting additional INMAD targets and studying ubiquitination/regulation of TMEM33 in metazoans, for example, will further our understanding of INM homeostasis across eukaryotes.

## Abbreviations

NPC: Nuclear pore complex
INM: Inner nuclear membrane
ONM: Outer nuclear membrane
NE: Nuclear envelope
NETs: Nuclear envelope transmembrane proteins
ER: Endoplasmic reticulum
INMAD: Inner nuclear membrane-associated degradation
ERAD: ER-associated degradation
Ub: Ubiquitin
SINC: Storage for incomplete NPCs
